# The development and validation of a numerical integration method for non-linear viscoelastic modeling

**DOI:** 10.1371/journal.pone.0190137

**Published:** 2018-01-02

**Authors:** Nicole L. Ramo, Christian M. Puttlitz, Kevin L. Troyer

**Affiliations:** 1 School of Biomedical Engineering, Colorado State University, Fort Collins, Colorado, United States of America; 2 Department of Mechanical Engineering, Colorado State University, Fort Collins, Colorado, United States of America; 3 Department of Clinical Sciences, Colorado State University, Fort Collins, Colorado, United States of America; Beihang University, CHINA

## Abstract

Compelling evidence that many biological soft tissues display both strain- and time-dependent behavior has led to the development of fully non-linear viscoelastic modeling techniques to represent the tissue’s mechanical response under dynamic conditions. Since the current stress state of a viscoelastic material is dependent on all previous loading events, numerical analyses are complicated by the requirement of computing and storing the stress at each step throughout the load history. This requirement quickly becomes computationally expensive, and in some cases intractable, for finite element models. Therefore, we have developed a strain-dependent numerical integration approach for capturing non-linear viscoelasticity that enables calculation of the current stress from a strain-dependent history state variable stored from the preceding time step only, which improves both fitting efficiency and computational tractability. This methodology was validated based on its ability to recover non-linear viscoelastic coefficients from simulated stress-relaxation (six strain levels) and dynamic cyclic (three frequencies) experimental stress-strain data. The model successfully fit each data set with average errors in recovered coefficients of 0.3% for stress-relaxation fits and 0.1% for cyclic. The results support the use of the presented methodology to develop linear or non-linear viscoelastic models from stress-relaxation or cyclic experimental data of biological soft tissues.

## Introduction

Viscoelastic theory describes the time-dependent relationship between stress and strain and is commonly used to describe the mechanical behavior of biological tissues. For viscoelastic materials, the current stress state is dependent upon all previous loading events. This history-dependent behavior complicates numerical analyses of viscoelastic materials because the stress at each step throughout the entire loading history must be computed and stored in order to obtain the current stress. For three-dimensional finite element models, computing and storing the stress tensor at each integration point and time step quickly becomes computationally intractable. To simplify numerical analyses for linear and quasi-linear viscoelastic materials, a discrete series of exponentials (such as a Prony series) is often used to approximate the continuous time-dependent relaxation spectrum.

As demonstrated by Puso and Weiss [1] for quasi-linear viscoelasticity (QLV), the unique properties of a discrete relaxation spectrum may allow for the current stress to be computed using only the stress from the previous time step, thereby greatly reducing computational expense. Fung’s theory of QLV [2,3] is a popular choice for researchers working with soft tissues due to its relatively straight-forward incorporation of hyperelastic formulations to describe elastic non-linearity. For example, it is widely used to described the behavior of connective (e.g., tendon [1,4] and ligament [1,5–7]) and spinal (e.g., spinal cord [8,9], brain [10], and dura mater [11]) tissues subjected to static and dynamic loading regimes. However, increasing evidence has demonstrated that these tissue types display fully non-linear viscoelasticity [12–17], wherein the non-linear elastic response cannot be separated from the non-linear time-dependent response.

The comprehensive viscoelastic characterization (CVC) method previously developed by our research group has been shown to accurately predict the non-linear viscoelastic cyclic response of both connective and spinal tissues based on fits of stress-relaxation data [13,18,19]. However, this technique is limited in three important ways: (1) it is restricted to fitting only stress-relaxation data, (2) it requires fits of individual stress-relaxation curves at each strain magnitude tested, and (3) it determines the strain-dependent behavior of the tissue *post-hoc* (from a subsequent fit of the strain-dependent behavior of the individual curve fits). To increase modeling flexibility and address each limitation above, the present study develops a novel numerical integration technique (called the *direct fit* method) for fully non-linear viscoelastic modeling. This novel methodology leverages the unique properties of the Prony series to allow the current stress to be computed from a deformation-dependent state variable stored from the preceding time step only. Similar to the formulation developed for QLV theory [1], the following non-linear viscoelastic formulation greatly improves computational tractability by avoiding the need to store the stress at each time step of the analysis. The following sections will present the derivation of our numerical integration technique, demonstrate its implementation using computational methods, and verify its ability to fit non-linear viscoelastic data by recovering a set of known non-linear viscoelastic coefficients. The significant advantage of a fully non-linear viscoelastic formulation over a linear viscoelastic formulation is also explicitly demonstrated through direct comparison of the fitting results.

## Materials and methods

### Model development

This section outlines the *direct fit* approach for non-linear viscoelastic modeling which calculates the current stress from a state variable stored from the preceding time step only (as opposed to every previous time step). A linear viscoelastic (i.e., strain-independent relaxation behavior) formulation follows the same derivation except where noted.

Uniaxial non-linear viscoelastic material behavior may be represented by the hereditary (or convolution) integral:

$$\sigma [\varepsilon (t),t]=\int_{0}^{t}E[\varepsilon (\tau ),t-\tau ]\frac{d\varepsilon (\tau )}{d\tau }d\tau ,$$ (1)

where *σ* is stress, *ε* is strain, *t* is time, *τ* is a time variable of integration representing the history effect, and *E*(*t*,*ε*) is the material relaxation modulus that describes the non-linear time-dependent relationship between stress and strain. The form of the relaxation modulus must be continuous and monotonically decreasing in order to satisfy thermodynamic restrictions [20]. When modeling biological tissues, it is common to approximate the continuous relaxation spectrum *E*(*t*,*ε*) by a discrete Prony series. For the case of non-linear viscoelasticity, the following strain-dependent Prony series has been shown to successfully capture the strain- and time-dependent behavior of several types of biological tissues [13,18,19,21]:

E[ε(t),t]=E∞(ε)+∑i=1NEi(ε)e−tτi, (2)

where *E*_*i*_(*ε*) is the strain-dependent Prony weight corresponding to time constant *τ*_*i*_, *E*_∞_(*ε*) represents the long-term strain-dependent modulus, and *N* defines the finite number of exponential Prony terms. For linear viscoelasticity, the Prony weights and long-term modulus are replaced with constant (strain independent) coefficients:

E(t)=E∞+∑i=1NEie−tτi. (3)

In order to satisfy the monotonically decreasing restriction on the relaxation modulus, the non-linear strain-dependent Prony weight functions must be positive and monotonically increasing (or a positive constant for linear viscoelasticity) and the time constants must be positive. Combining Eq (1) and Eq (2) yields the following definition for stress at the current time *t*, assuming *ε*(0) = 0:

σ[ε(t),t]=∫0t{E∞(ε)+∑i=1NEi(ε)e−(t−τ)τi}dε(τ)dτdτ=∫0tE∞(ε)dε(τ)dτdτ+∫0t{∑i=1NEi(ε)e−(t−τ)τi}dε(τ)dτdτ=E∞(ε)[ε(t)−ε(0)]+∫0t{∑i=1NEi(ε)e−(t−τ)τi}dε(τ)dτdτ=E∞(ε)ε(t)+∫0t{∑i=1NEi(ε)e−(t−τ)τi}dε(τ)dτdτ (4)

A strain-dependent history state variable is defined to recursively update the stress at each incremental time step:

hi[ε(t),t]=∫0t{Ei(ε)e−(t−τ)τi}dε(τ)dτdτ, (5)

such that Eq (4) can be recast as:

$$\sigma [\varepsilon (t),t]=E_{\infty }(\varepsilon )\varepsilon (t)+\sum_{i=1}^{N}h_{i}[\varepsilon (t),t].$$ (6)

The stress at the next time step, *t* + Δ*t*, is given as:

$$\sigma [\varepsilon (t+\Delta t),t+\Delta t]=E_{\infty }(\varepsilon )\varepsilon (t+\Delta t)+\sum_{i=1}^{N}h_{i}[\varepsilon (t+\Delta t),t+\Delta t],$$ (7)

where the updated history variable is:

hi[ε(t+Δt),t+Δt]=∫0t+Δt{Ei(ε)e−(t+Δt−τ)τi}dε(τ)dτdτ. (8)

Eq (8) can be expanded by use of the summation rule for definite integrals:

hi[ε(t+Δt),t+Δt]=∫0t{Ei(ε)e−(t+Δt−τ)τi}dε(τ)dτdτ+∫tt+Δt{Ei(ε)e−(t+Δt−τ)τi}dε(τ)dτdτ. (9)

Inputting Eq (9) into Eq (7) yields the following expression for the stress at the next time step:

σ[ε(t+Δt),t+Δt]=∑i=1N∫0t{Ei(ε)e−(t+Δt−τ)τi}dε(τ)dτdτ+∑i=1N∫tt+Δt{Ei(ε)e−(t+Δt−τ)τi}dε(τ)dτdτ+E∞(ε)ε(t+Δt), (10)

where the first term represents the history effect (integrated over all previous loading events), the second term represents the effect of the current loading event, and the final term represents the effect of the equilibrium response.

Using the product law of exponentials, the history state variable, *h*_*i*_[*ε*(*t*),*t*], could be factored out of the first term of Eq (9):

∫0t{Ei(ε)e−(t+Δt−τ)τi}dε(τ)dτdτ=hi[ε(t),t]{∫0t{Ei(ε)e−(t+Δt−τ)τi}dε(τ)dτdτ∫0t{Ei(ε)e−(t−τ)τi}dε(τ)dτdτ}=hi[ε(t),t]{e−tτie−Δtτi∫0t{eττi}dε(τ)dτdτe−tτi∫0t{eττi}dε(τ)dτdτ}=hi[ε(t),t]e−Δtτi. (11)

The second mean-value theorem of integrals states that for continuous functions *f*(*x*) and *g*(*x*) ≥ 0 over *x* ∈ [*a*,*b*], there exists *c* ∈ (*a*,*b*) such that $\int_{a}^{b}f(x)g(x)dx=f(c)\int_{a}^{b}g(x)dx$. This theorem is imposed on the second term of Eq (9) such that:

∫tt+Δt{Ei(ε)e−(t+Δt−τ)τi}dε(τ)dτdτ=dε(k)dτ∫tt+Δt{Ei(ε)e−(t+Δt−τ)τi}dτ, (12)

with *k* ∈ (*t*,*t* + Δ*t*). The time steps are assumed to be small enough that the error associated with linear interpolation between sequential strain values is negligible. Accordingly, by the central difference rule:

$$\frac{d\varepsilon (k)}{d\tau }=\frac{\varepsilon (t+\Delta t)-\varepsilon (t)}{t+\Delta t-t}=\frac{\Delta \varepsilon }{\Delta t}.$$ (13)

Using Eq (13), the second term of Eq (9) may be evaluated as:

ΔεΔt∫tt+Δt{Ei(ε)e−(t+Δt−τ)τi}dτ=Ei(ε)ΔεΔt{τie−(t+Δt−τ)τi|τ∈[t,t+Δt]}=Ei(ε)τiΔεΔt(1−e−Δtτi). (14)

Therefore, Eq (9) may be simplified as:

hi[ε(t+Δt),t+Δt]=hi[ε(t),t]e−Δtτi+Ei(ε)τiΔεΔt(1−e−Δtτi). (15)

and Eq (7) can be recast as:

σ[ε(t+Δt),t+Δt]=E∞(ε)ε(t+Δt)+∑i=1N{hi[ε(t),t]e−Δtτi+Ei(ε)(1−e−Δtτi)(Δtτi)Δε}. (16)

Using the incremental notation *f*_*n*+1_ = *f*_*n*_ + Δ*f*_*n*_, where *f* is an incremental variable, *f*_*n*_ is the variable value at the preceding increment, and Δ*f*_*n*_ is the current variable increment, the following incremental formulation for non-linear viscoelasticity is obtained:

σ(εn+1)n+1=E∞(ε)εn+1+∑i=1N{hi[ε(t),t]e−Δtnτi+Ei(ε)(1−e−Δtnτi)(Δtnτi)Δεn+1}. (17)

Following similar mathematical development, the analogous equation for linear viscoelasticity is:

σn+1=E∞εn+1+∑i=1N{hi(t)e−Δtnτi+Ei(1−e−Δtnτi)(Δtnτi)Δεn+1}. (18)

It should be noted that evaluating Eq (17) or Eq (18) at the current time step requires only the history state variable from the previous time step (*h*_*i*_[*ε*(*t*),*t*] for non-linear viscoelasticity and *h*_*i*_(*t*) for linear viscoelasticity). Unlike the CVC method previously developed by our group [13,19], the presented formation may be fit to an arbitrary strain history and may be used in a data fitting algorithm to directly determine the non-linear strain-dependence of each Prony weight.

### Model validation

The numerical integration technique for our *direct fit* method, and its associated non-linear viscoelastic model, were validated based on its ability to recover coefficients used to create idealized experimental data. These stress-strain data were created by specifying the mathematical formulae and coefficients of the non-linear relaxation modulus (*E*[*ε*(*τ*),*t*]), the associated time constants, and the strain magnitude. Values were chosen based on the experimental data provided in Troyer et al. for ovine Achilles tendon [19]. In this previous work, the non-linear viscoelastic relaxation modulus was approximated by a 4-term Prony series, where each strain-dependent Prony weight was described with a two-term polynomial function:

$$E_{i}(\varepsilon )=C_{1}^{\tau_{i}}\varepsilon +C_{2}^{\tau_{i}}\varepsilon^{2},$$ (19)

$$E_{\infty }(\varepsilon )=C_{1}^{\infty }\varepsilon +C_{2}^{\infty }\varepsilon^{2}.$$ (20)

The time constants were fixed at decadal values (*τ*_1_ = 0.1 s, *τ*_2_ = 1 s, *τ*_3_ = 10 s, *τ*_4_ = 100 s) in order to adequately capture both the short-term and long-term response of the tissue. The *C*_1_ and *C*_2_ coefficients obtained via the CVC method (Table 1, [19]) were input into Eq (17) to create idealized experimental data for two types of viscoelastic experiments: static stress-relaxation and dynamic cyclic tests. The idealized experimental data of each type were then simultaneously fit, in their entirety, to both the presented non-linear and linear viscoelastic models using MATLAB’s (R2014b, Mathworks, Natick, MA) *fmincon* constrained non-linear optimization function. For the non-linear viscoelastic fits, each Prony weight was constrained to be positive and monotonically increasing in order to satisfy thermodynamic restrictions. For the linear viscoelastic fits, the Prony constants were constrained to be positive. Since multiple curves were fit simultaneously, the root mean squared errors (RMSEs) for individual curves in the fit were summed and used to define the objective function minimized by the MATLAB algorithm.

**Table 1:** Input and recovery error of non-linear viscoelastic coefficients.

	Input Coefficients [19] (MPa)	Stress-Relaxation (n = 6) Coefficient Recovery Error	Dynamic Cyclic (n = 3) Coefficient Recovery Error
$C_{1}^{\tau =0.1}$	**901.1**	0.01%	0.003%
$C_{2}^{\tau =0.1}$	**8437**	0.05%	0.01%
$C_{1}^{\tau =1}$	**343.5**	0.16%	0.01%
$C_{2}^{\tau =1}$	**-684.1**	2.18%	0.12%
$C_{1}^{\tau =10}$	**331.2**	0.03%	0.01%
$C_{2}^{\tau =10}$	**-1201.2**	0.24%	0.08%
$C_{1}^{\tau =100}$	**476.5**	0.002%	0.02%
$C_{2}^{\tau =100}$	**-363.4**	0.07%	0.90%
$C_{1}^{\infty }$	**4403.1**	0.0001%	0.001%
$C_{2}^{\infty }$	**-9959.3**	0.001%	0.01%
**Average**		**0.28%**	**0.12%**

The proposed numerical integration *direct fit* method for non-linear viscoelastic characterization was able to recover input non-linear viscoelastic coefficients using both stress-relaxation and dynamic cyclic stress-strain data with average errors well below 1%.

The accuracy of model fits were assessed by computing the RMSE between each idealized experimental stress-strain curve and that predicted by each viscoelastic model. For the non-linear viscoelastic model, the recovery of input *C*_1_ and *C*_2_ coefficients was assessed by the percent error for each of the 10 coefficients.

## Results

### Stress-relaxation

**Fig 1:**
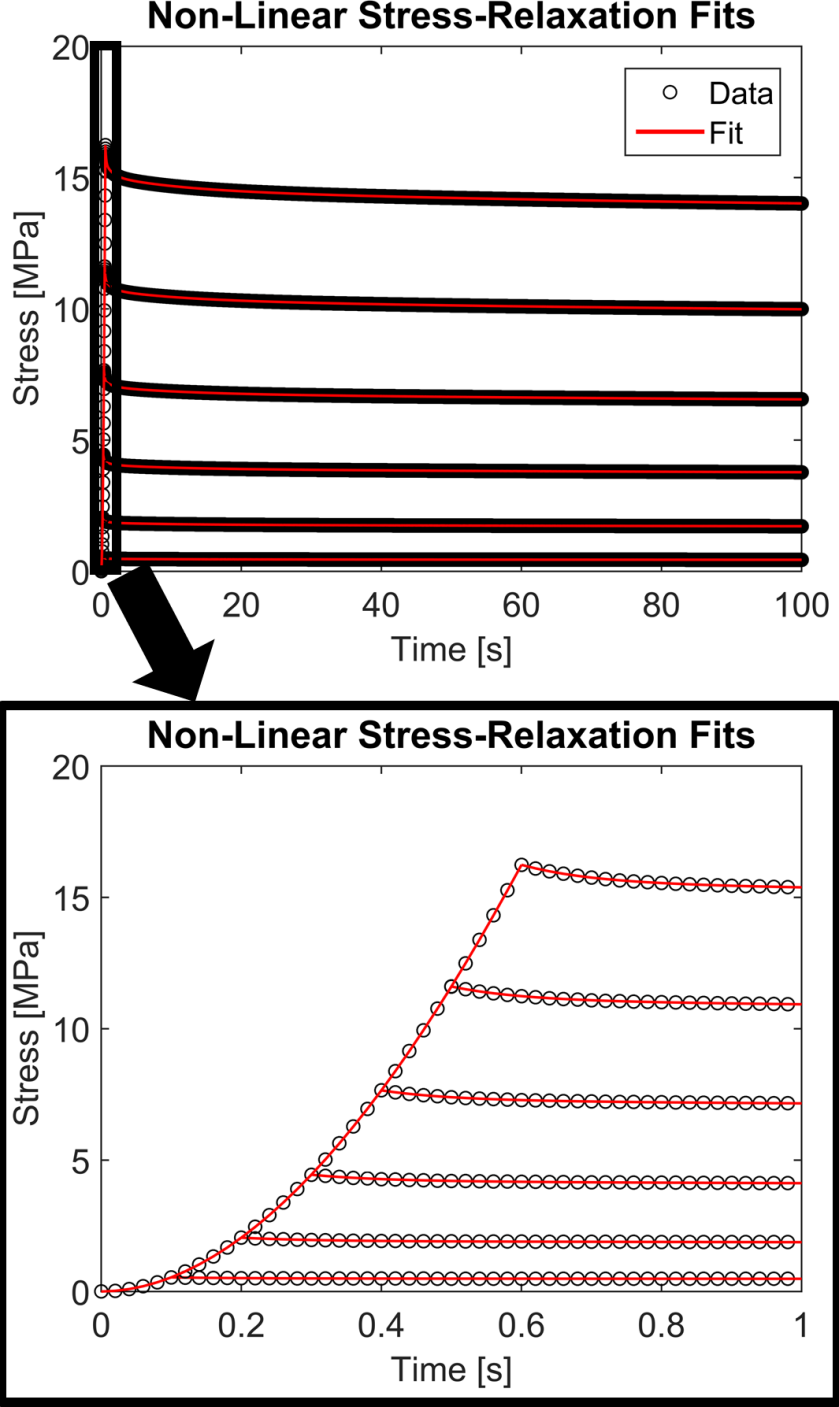
Non-linear stress-relaxation fits. The proposed numerical integration *direct fit* method for non-linear viscoelastic characterization was able to accurately fit the idealized stress-relaxation experimental data, including the non-linear stress-strain behavior during the ramping phase and the strain-dependent relaxation indicative of non-linear viscoelastic behavior.

Six idealized stress-relaxation experimental curves were created to match the experimental work by Troyer et al. [19]. Specifically, stress-relaxation experiments at 1%, 2%, 3%, 4%, 5%, and 6% engineering strain at a ramping strain-rate of 0.1/sec with a dwell time of 100 seconds were created using the coefficients in Table 1. An initial guess value of 100 was used for all ten fitted coefficients in the simultaneous fits of the six experimental curves. As shown in Fig 1, the non-linear viscoelastic model fit the idealized experimental curves very well, including the non-linear stress-strain behavior during the ramping phase, with RMSE values ranging from 1.4 to 12.5 Pa (average RMSE = 5.25 Pa). These RMSE values are approximately six orders of magnitude less than the peak stress, representing less than 0.003% of the peak stress. On average, there was less than a 0.28% difference between the fitted coefficients and the coefficients used to create the experimental curves (range 0.0001% to 2.18%, Table 1).

**Fig 2:**
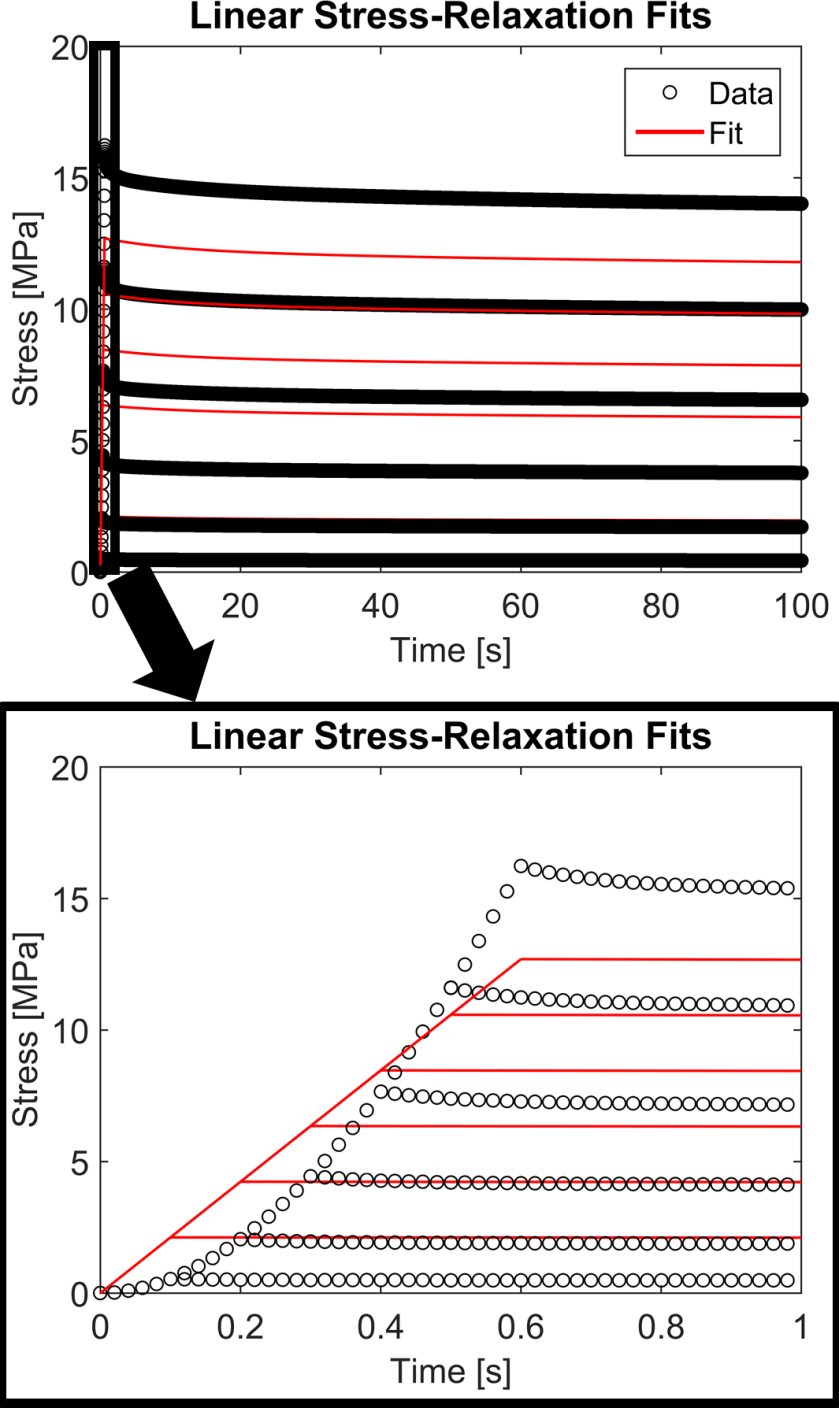
Linear stress-relaxation fits. The linear viscoelastic formulation was not able to capture the idealized strain-dependent stress-relaxation data, resulting in large RMSE values compared to those of the non-linear viscoelastic formulation.

Contrary to the non-linear viscoelastic model, the linear viscoelastic model was unable to describe the strain-dependent stress-relaxation data. As shown in Fig 2, the linear model could not capture the non-linear stress-strain behavior during the ramping phase nor the non-linear strain-dependent relaxation response. The RMSE values for the linear model fit were up to six orders of magnitude larger than the values obtained for the non-linear model fit. The RMSE values for the linear model fit ranged from 0.20 to 2.27 MPa representing an average 80% of the peak stress.

### Dynamic cyclic

The ability of the *direct fit* method to recover input coefficients from dynamic cyclic data was also examined. Similar to the stress-relaxation methodology, idealized experimental data were created using the same time constants and relaxation modulus coefficients obtained in Troyer et al. [19] (Table 1). Three idealized experimental dynamic cyclic data curves consisting of 10 cycles to the maximum strain level of interest (6%) at 0.01Hz, 0.1Hz, and 1Hz were created for fitting. As with the stress-relaxation fits, an initial guess of 100 was used for all ten coefficients in the simultaneous fits of the three curves.

**Fig 3:**
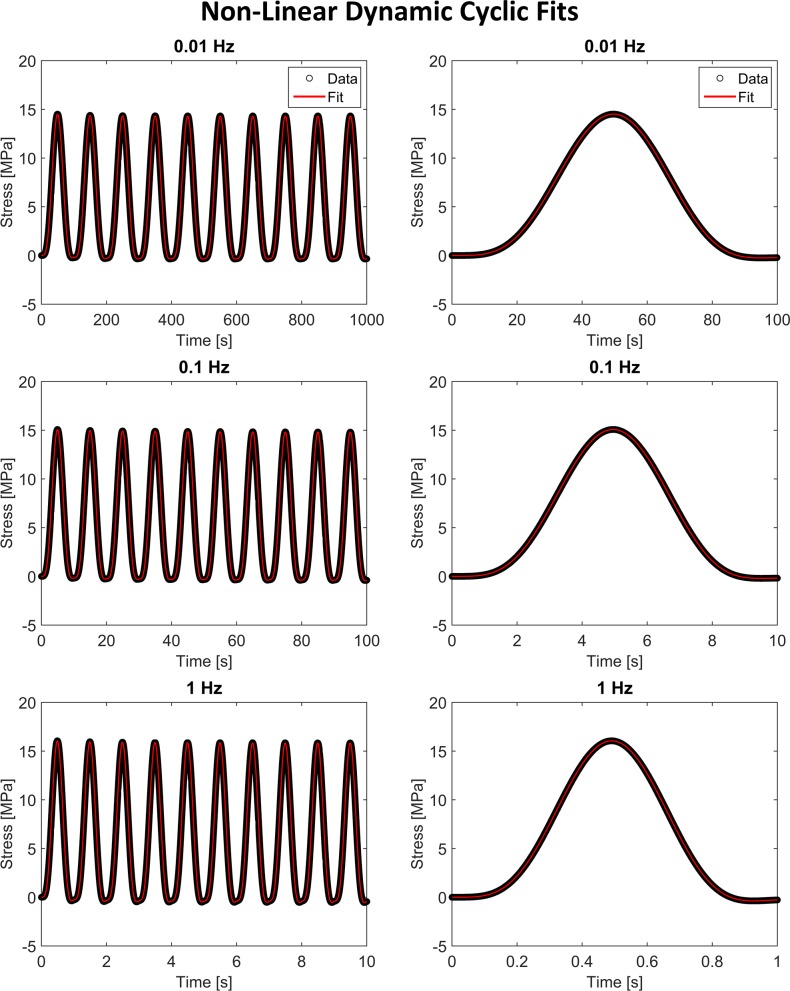
Non-linear dynamic cyclic fits. The proposed *direct fit* method accurately fit the idealized dynamic cyclic response at three frequencies. These curves were fit simultaneously but are plotted separately to improve visualization of the higher frequency fits. Images in the right column show the first cycle of the fit for each frequency.

The non-linear viscoelastic model also fit the cyclic data very well, with RMSE values (2.75, 2.24, and 1.66 Pa) almost seven orders of magnitude less than the peak stress (Fig 3). The cyclic fits also exhibited strong coefficient recovery with an average 0.12% difference between the fitted coefficients and the coefficients used to create the idealized experimental curves (range 0.001% to 0.9%, Table 1).

**Fig 4:**
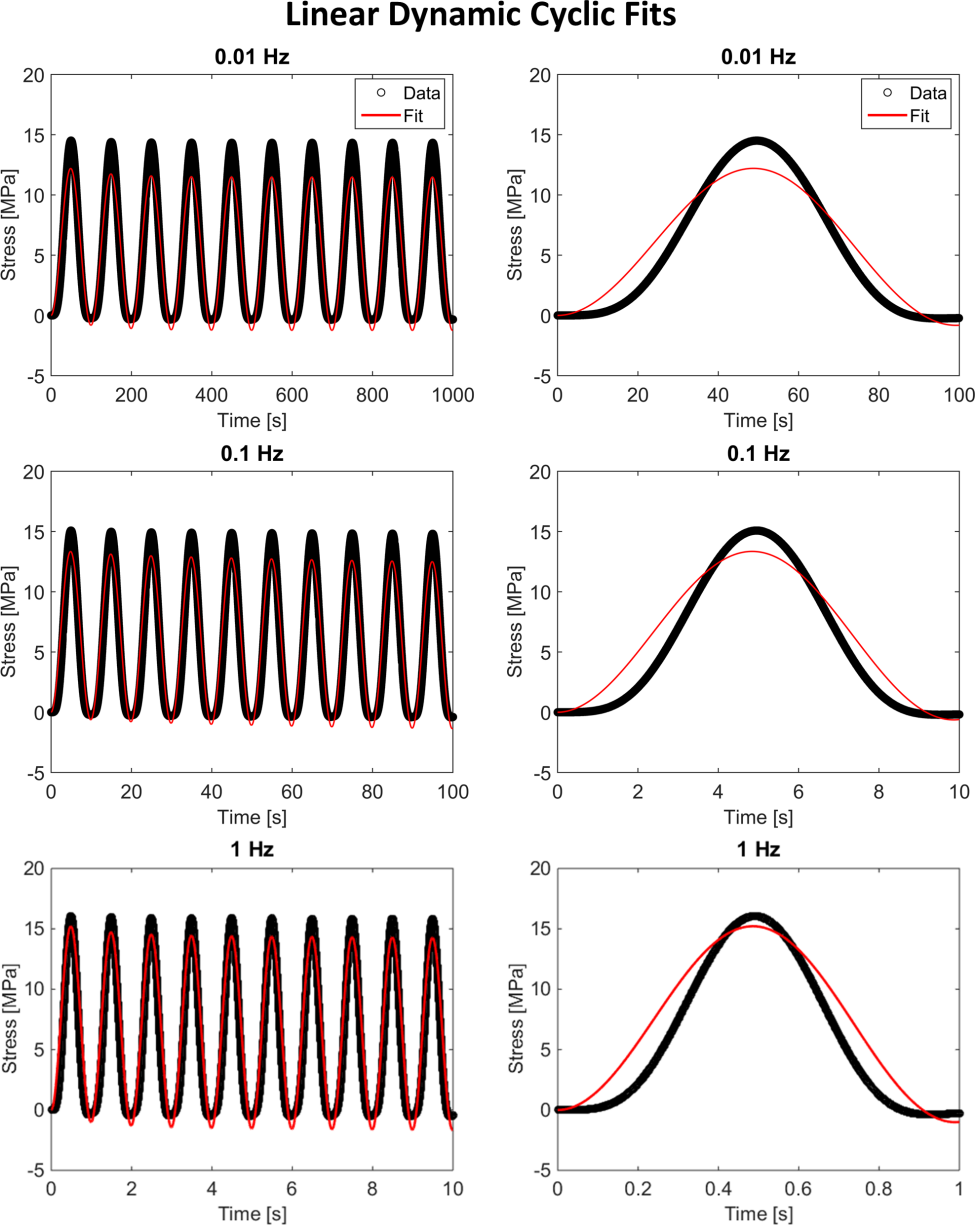
Linear dynamic cyclic fits. The linear viscoelastic formulation was unable to fit the idealized dynamic cyclic response, resulting in very large RMSE values compared to those of the non-linear viscoelastic formulation. These curves were fit simultaneously but are plotted separately to improve visualization of the higher frequency fits. Images in the right column show the first cycle of the fit for each frequency.

Fig 4 shows the results of fitting the linear viscoelastic model to the same three dynamic cyclic curves. As with the stress-relaxation data, the linear viscoelastic model was unable to capture the strain-dependent viscoelastic response with RMSE values six orders of magnitude larger than those obtained for the non-linear model fit. The linear model resulted in RMSE values of 1.44, 1.47, and 1.65 MPa, which represents approximately 10% of the peak stresses.

## Discussion

With increasing experimental evidence that the mechanical behavior of many biological tissues is not adequately captured by linear and quasi-linear viscoelastic formulations, there is a significant need for computationally tractable fully non-linear viscoelastic modeling methods. The novel *direct fit* method presented herein provides a number of advantages over other non-linear techniques, including that of the CVC method [13,18,19]. Specifically, through the use of a strain-dependent Prony series representation of the relaxation modulus and the product law of exponentials, the *direct fit* method does not require storage of the stress at each time step of the loading history. Instead, the new method recursively updates a strain-dependent history state variable. The new method also permits simultaneous fits of all experimental data from each sample, which is believed to result in a better approximation of the sample’s behavior than averaging the results of individual curve fits. In addition, by fitting the data curves in their entirety, the non-linearity is directly determined from the fits themselves instead of *post hoc* analyses (as with the CVC method). Finally, the *direct fit* method also allows for more experimental flexibility since it may be fit to an arbitrary strain history (e.g., stress-relaxation and cyclical experiments or combinations thereof). While the CVC method is both efficient in its fits and accurate in its predictions [13,19], it is limited to fitting the stress-relaxation response only. Since non-linear viscoelastic characterization based on stress-relaxation data require multiple tests at varying strain magnitudes, this experimental procedure can require significant experimental testing time. For tissues whose mechanical properties demonstrate a relatively quickly post-mortem degradation profile, such as neural tissues [22–24], the ability to fit fewer cyclic experiments for the same predictive accuracy is a very important advantage.

Strong recovery of all ten input coefficients from both stress-relaxation and cyclic experimental data validates the use of the *direct fit* method for non-linear viscoelastic characterization. It is important to note that, on average, the cyclic fits were better at recovering the input coefficients than the stress-relaxation fits. As the strain history is continuously changing over the course of the test, the strain-dependent Prony weights are more sensitive when fitting cyclic data compared to strain-stagnant relaxation data.

While the non-linear viscoelastic model fit the idealized experimental data very well, the linear viscoelastic model performed much worse with RMSE values of up to 200% of the peak stress. The inability of the simplified linear viscoelastic model to fit the idealized experimental data demonstrates the need for fully non-linear viscoelastic models to characterize the mechanical behavior of many biological tissues. As seen in Figs 2 and 4, the linear formulation of the presented model was unable to capture non-linear elastic or non-linear viscous behavior, both of which are commonly seen in the mechanical response of connective and neural tissues [12–17].

Limitations of the *direct fit* method developed herein include the restriction to uniaxial tension and the use of a simple polynomial model to capture the strain-dependent Prony weights. While uniaxial tension tests are popular experimental methods for characterizing both connective and neural tissues, we plan to extend the method to include descriptions of anisotropic behavior by investigating strain energy based formulations to describe the strain-dependence of the Prony weights. In future work, we will use the numerical integration *direct fit* technique to characterize the viscoelastic behavior of spinal cord and meningeal tissues in order to improve the time-dependent mechanical behavior predictions of spinal cord injury finite element models.
